# The role of the Nocebo effect in the use of biosimilars in routine rheumatology clinical practice

**DOI:** 10.31138/mjr.30.1.63

**Published:** 2019-05-31

**Authors:** Evrydiki Kravvariti, George D. Kitas, Petros P. Sfikakis

**Affiliations:** 1 First Department of Propaedeutic Internal Medicine, Joint Rheumatology Program, National & Kapodistrian University of Athens Medical School, Athens, Greece,; 2 Clinical Research Unit, Dudley Group NHS Foundation Trust, Dudley, UK,; 3 Arthritis Research UK Centre for Epidemiology, University of Manchester, Manchester, UK

**Keywords:** Nocebo, nocebo effect, biosimilars, rheumatoid arthritis, drug discontinuation

## NOCEBO DEFINITIONS AND PATHOPHYSIOLOGIC MECHANISMS

The management of patients with rheumatic diseases in routine practice is guided by established measures of response to treatment with a target of low disease activity or remission. In addition to objective clinical data, subjective patient-reported outcomes (such as the number of tender joints or the patient’s perception of their general health status) are taken into account when assessing efficacy and tolerability of disease-modifying anti-rheumatic drugs (DMARDs), including bio-originator and biosimilar biologic DMARDs. A growing body of evidence suggests that the impact of DMARDs on subjective symptoms in rheumatic diseases is significantly affected by the patients’ expectations of treatment effects, either favorably when pre-existing expectations are positive (placebo effect), or detrimentally when pre-existing expectations are negative (nocebo effect).^1–4^

The word nocebo (from Latin noceo, meaning “to harm”) denotes a medical intervention (a medication, inert substance, procedure, or patient-physician encounter) that causes noxious changes in a patient’s symptoms or physiologic condition because of negative expectations.^5,6^ These nocebo effects may manifest as new adverse symptoms, or as a recurrence of unpleasant symptoms previously experienced in the setting of a chronic disease. Nocebo effects often mimic the side effects described in clinical trial consent forms or patient information leaflets, but may also be nonspecific subjective complains, such as dizziness, fatigue, or pain. Neurobiological research based on functional MRI imaging has shown that nocebos activate specific areas in the modulatory pain network, which may result in exacerbation of the patient’s pain or even generate the perception of a pain signal in the patient’s brain. These areas include parts of the limbic system linked with anxiety and emotional response to stress (hippocampus, amygdala), centers for decision-making, reward, and survival behavior in response to threat (insula, nucleus accumbens, anterior cingulate cortex), and their interconnections with the periaqueductal grey and rostral ventral medulla, which modulate ascending pain signals.^7–11^ By facilitating nocebo effects and materializing negative preconceptions, these neural circuits may promote risk avoidance and learning from other people’s adverse experience.^12^ Additionally, relevant studies show that nocebo circuits manifest neuronal plasticity and increased activity as a result of prior conditioning with adverse drug effects, stressful situations and co-existing anxiety disorders.^10,13^

## NOCEBO RISK FACTORS

**Figure 1:**
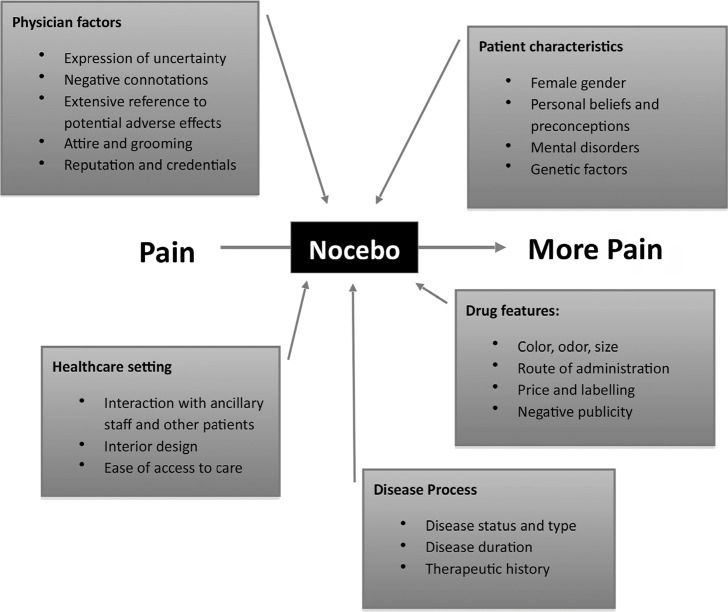
Nocebo risk factors in clinical practice.

Experimental and translational research has identified several risk factors for the development of nocebo effects, most of which are very prevalent among patients with rheumatic disease. These risk factors can be classified as physician attributes, patient characteristics, drug-related factors, features of the healthcare setting, and factors related to the disease process (*Figure 1*).^5^ Among these, the interaction between patient and physician at the time of the medical encounter has the most catalytic impact on the development of nocebo effects. Nocebos affect patients of all ages, but a recent meta-analysis suggests women are more susceptible than men.^14^ Patients with somatisation, depression and anxiety disorders, chronic pain and pain catastrophizing, cognitive impairment, or language barriers all predispose patients to nocebo effects.^15–18^ History of adverse effects to prior treatment, and a recurrently flaring disease course with failure to multiple lines of therapy results in negative conditioning, also leading to nocebo effects.^3^ Refusal of interventions, such as preventive vaccination, and/or extensive online researching may be indications of deep apprehension and negative bias toward medications.

On behalf of the physician, expressing uncertainty or anxiety about the proposed intervention, using negative connotations and framing while informing patients, or overtly focusing on potential adverse effects using lengthy and vivid descriptions, has been linked to a higher rate of nocebo effects.^19–21^ A dismissive communication style (including language, posture, and grimace), as well as unbefitting comments, attire, and grooming, will affect patient expectations, both consciously and subconsciously. Finally, other features of the healthcare setting may predispose patients to nocebo effects, such as architecture, interior design, technology, accessibility and affordability of care, and behaviour of non-physician staff. Patients who spend a lot of time in waiting rooms and infusion suites may be exposed to negative suggestions and groupthink effects as a result of discussions with fellow patients.

## NOCEBO EFFECTS IN PATIENTS WITH RHEUMATIC DISEASE

It is known that patients with rheumatic disease and chronic pain have altered pain transmission and central sensitization even after the inflammatory aspect of their pain has resolved.^22^ It is estimated that up to 30% of rheumatoid arthritis (RA) patients achieving low disease activity or remission targets with biologic DMARDs experience residual non-inflammatory pain, which can be attributed to impaired pain modulation.^2^ Patients with fibromyalgia syndrome suffer primarily due to impaired pain signal inhibition.^23^ Functional brain imaging evidence suggests that in osteoarthritis, changes in the peripheral and central processing of ascending pain signals may be largely responsible for the difficult-to-treat chronic pain cases.^24^ Due to its subjective nature and prevalence as a cardinal symptom of arthritis, pain increase is an expected nocebo-related adverse outcome in patients with rheumatic diseases.

Although nocebo may occur commonly in routine rheumatology practice, it can be hard to discern whether an unfavourable response to therapy is a pharmacological adverse event, a result of spontaneous fluctuations in disease activity, or a nocebo effect. In the randomized clinical trial (RCT) context, nocebo effects are measured as adverse events occurring in the inert substance arm and classified as “drug-related” by the blinded investigators. Meta-analyses of inert substance arm dropouts from RCTs for rheumatic diseases have shown that dropouts due to nocebo effects are more prevalent in patients with fibromyalgia syndrome (∼10% in trials of analgesic therapies), as compared to patients with RA (∼3% in trials of bio-originator biologic DMARDs and relevant meta-analyses of analgesic trials).^5,25,26^ On the other hand, trial participants with RA, have higher rates of inert substance discontinuations due to lack of efficacy (7% in a recent meta-analysis) compared to participants with chronic neuropathic pain (3%) or osteoarthritis (1%), possibly hinting to a relative “resistance” to the placebo effect.^26^

## NOCEBO AND BIOSIMILARS

In recent years, biosimilar biologic DMARDs (BSM) emerged as a preferred treatment for patients with a variety of rheumatic diseases over bio-originator biologic DMARDs (BO), because they are marketed at a significantly lower price, and switching patients from BO to BMS agents could result in significant healthcare fund savings (estimates reach £200 million/year for the NHS).^27^ Despite affirming results from several double-blind RCTs examining bio-equivalence and non-inferiority following open-label switch to a BSM versus remaining on the BO,^28–33^ results from nationwide switch observational studies and other real-world cohorts revealed alarming drug discontinuation rates of 15–30%, mostly for perceived lack of efficacy and subjective non-specific complains without increase in inflammatory markers.^4,34–41^

In a meta-analysis of all published BSM switch studies through 2018, the pooled drug discontinuation rate for 21 open-label studies (both RCT and real-world observational studies) was significantly higher than observed in 2 double-blind RCTs (15% vs. 7%).^27^ This discrepancy has been interpreted as a nocebo effect, adding to the long list of suboptimal outcomes associated with nocebo.^42,43^ It should be noted though, that other reasons may also play a role, such as a selection of biologic-naïve patients for RCTs, a less tight follow-up in the real-world setting, and causal misattributions on behalf of the physicians, who may discontinue BSMs in response to non-specific subjective complains more frequently that they would if the patient were on a BO.^5^ In the BIO-SWITCH multicentre prospective open-label study from the Netherlands, the investigators noted that although adverse events in the arm treated with BSMs was the same as in the blinded NOR-SWITCH from Norway (18 vs. 10%), the lead to BSM discontinuation more often (11% vs. 3%).^4,28^ This finding is congruent with negative preconceptions and uncertainty of prescribing physicians about the bio-equivalence of BSMs, which has been recorded in relevant studies.^27,44–46^

It is expected that patients with rheumatic disease will be even more prone to negative preconceptions about BSMs than physicians, due to previous adverse drug reactions and inability to interpret scientific medical data. Patients regard their rheumatologist as the most influential source of information and the most important contributor to agreeing to transition to a biosimilar, but objective and thorough patient education on BSMs seems to be lacking: in one study from Europe, scientific information on BSMs was inadequately provided to patients treated or about to be treated with a biosimilar, even though having a good understanding of BSMs lead to better adherence.^46^

## SUGGESTIONS FOR CLINICAL PRACTICE

Clinical consequences of nocebo effects are far-reaching and include medication non-adherence and wasting, over-utilization of healthcare resources, polypharmacy, loss of patient trust, treatment with second-line therapies and suboptimal outcomes, and dropouts from clinical trials hindering interpretation and generalization of medical research findings.^3,43,47–49^ Assuming a rate of nocebo-related drug discontinuation of 3–10% supported by relevant literature in patients with rheumatic disease,^5,27,41^ implementation of strategies to diminish nocebo effects could result in higher rates of successful initial treatments and BSM transitions. Both individual physicians and healthcare organization administrators must take action to limit the impact of nocebo effects on clinical outcomes, focusing on providing patient education and addressing patient concerns.

Physicians must be able to identify patients with many risk factors for nocebo effects, in order to allocate more time for counselling and educating them about nocebo: it has been shown that high-risk patients who understand nocebo mechanisms are less likely to develop nocebo effects.^50^ Several questionnaires have been used in research settings to assess negative beliefs about medications (Q-No,^51^ Stanford Expectations of Treatment Scale,^52^ Beliefs About Medications53 ); however, more research is needed in order to identify a scale with satisfactory sensitivity and specificity for the prediction of nocebo effects. Regardless of specific tools used, healthcare professionals should take the time to elicit patient’s beliefs about treatment, prior history of adverse effects, and other stressful situations hindering adherence to medications.

When physicians suspect medication intolerance due to non-specific, pharmacologically implausible complains in high-risk patients, it may be worth attempting causal re-attributions or positive expectation induction, which has been shown to diminish and even reverse nocebo effects;^50,54^ frequent visits may be needed if patients are agreeable. A change in the time or place the patients take their medication may be warranted, if negative associations are identified. Physicians may also advise nocebo-prone patients to ignore patient information leaflets and provide individualized information on side effects, using graphic illustrations and positive framing. In a recent RCT, stating that a side effect was “uncommon, with 90% of people unaffected” with an inert substance led to 39% nocebo effects, versus 55% in the group where the same side effect was presented as “common, with 1 in 10 people affected”.^55^

Effective and positive patient-physician interaction is a key element toward the prevention of nocebo effects in all patients. Physicians who generate fear, dismiss patient concerns or seem emotionally unavailable cause negative patient anticipation; those who provide reassurance, show empathy and empower patients during their encounters forge a strong therapeutic alliance. Phrasing that avoids negative connotations and stresses expected benefits rather than safety concerns should be preferred in decision-making discussions and consent forms. Discussions about drug cost require caution, as patients tend to consider low-cost alternatives either less safe or less effective.^56–58^ Behavioural science data supports that face-to-face delivery of information provides stronger re-assurance than written material alone,^16^ which should be taken into account in appointment scheduling. “Soft skills” training aimed at enabling rheumatologists to maximize placebo effects and minimize nocebo effects should be applied, not only because it will make physicians and patients “feel better”, but in order to bring about a measurable positive impact on health outcomes for our patients.
